# The International Medical Annual and Practitioners’ Index for 1892

**Published:** 1892-06

**Authors:** 


The International Medical Annual and Practitioner's Index for 1892. Edited by P. W. Williams, M. D., Secretary of Staff, assisted by a corps of thirty-two collaborators, European and American, specialists in their several departments. Six hundred and forty-four octavo pages. Illustrated. $2.75. E. B. Treat, publisher, 5 Cooper Union, New York.

This decidedly popular and valuable reference book is now before the public, and certainly the tenth issue is much superior to any of its predecessors. The corps of department editors have spared no pains in bringing their special fields up to the latest period before going to press, thus informing the busy practitioner of the most recent advances in our science.

The Annual is divided into three parts. Part I. treats of New Remedies, and New Applications of Old Remedies. Such agents as spermine, strontium, cactus, etc., are fully described, together with their therapeutic indications. Part II. comprises the major portion of the book, and is given to the consideration of New Treatment. Part III. contains short papers by well-known writers on subjects of great importance to medical men. Those on The Recent Advances in Bacteriology, Medical Photography, Sanitary Science, and Use of Suppositories, show the range of subjects. The appendix contains a list of the medical works published during the year ; also an incomplete list of new instruments, and a list of medical publishers. The illustrations, especially those in colors, as the endoscopic appearances of the bladder, etc., are handsomely executed, and add to the beauty of the Annual. Although everything is arranged in alphabetical order, a complete index is added, making it possible to seek any desired information with the greatest saving of time.

On the whole, the Annual is a very practical and handy book to possess, while, at the same time, the cost is trifling. W. C. K.

